# Curcumin in oral potentially malignant disorders and oral squamous cell carcinoma: Clinical and biological evidence from a systematic review

**DOI:** 10.4317/jced.64298

**Published:** 2026-08-28

**Authors:** Andrea Maturana-Ramírez, Gabriel Rojas-Zúñiga, Cristóbal Soza Cisternas, Juan Aitken-Saavedra

**Affiliations:** 1 Department of Oral Pathology and Oral Medicine, Faculty of Dentistry, Universidad de Chile, Santiago, Chile; 2 Faculty of Dentistry, Universidad de Chile, Santiago, Chile

**Keywords:** Oral leukoplakia, oral lichen planus, curcumin, mouth neoplasms.

## Abstract

**Background:**

Oral squamous cell carcinoma is associated with poor survival and frequently develops from oral potentially malignant disorders, particularly oral leukoplakia and oral lichen planus. Conventional management is mainly based on the removal of clinically altered areas; however, the risk of progression may persist. In this context, curcumin, a natural compound derived from turmeric with antioxidant, anti-inflammatory, and antineoplastic properties, has been investigated as a potential therapeutic alternative.

**Materials and Methods:**

A qualitative systematic review was conducted following the Cochrane Handbook and PRISMA guidelines. Literature was searched in MEDLINE (PubMed), Web of Science, Scopus, and SciELO (September-December 2025), including human studies from the last 15 years in English, Spanish, or Portuguese. Studies on adult patients with oral leukoplakia, oral lichen planus, or oral squamous cell carcinoma treated with curcumin or Curcuma longa derivatives (oral or topical) were included. Outcomes assessed included clinical efficacy (lesion size and symptoms), anti-inflammatory effects, and molecular biomarkers.

**Results:**

A total of 1,527 articles were identified, of which 18 met the inclusion criteria, comprising 1,189 participants. Most studies were clinical trials. In oral leukoplakia, all studies reported clinical improvement, mainly reflected by lesion size reduction, although without consistent histopathological changes. In oral lichen planus, eight of ten studies showed reductions in pain and lesion size, with decreases in VAS scores and statistically significant differences (p<0.05). Relevant reductions in lesion size were also observed (p<0.05). In oral squamous cell carcinoma, a significant decrease in inflammatory cytokines (IL-1β, IL-8, TNF-α) was reported. Comparable efficacy to corticosteroids was observed in most comparative studies.

**Conclusions:**

The use of curcumin appears to provide favorable clinical effects and relevant anti-inflammatory benefits in oral leukoplakia, oral lichen planus, and oral squamous cell carcinoma, mainly reflected by reductions in pain and lesion size. However, further well-designed clinical trials are required to confirm its efficacy and to better define its role in clinical practice.

## Introduction

Oral squamous cell carcinoma (OSCC) is the most common malignancy of the oral cavity and may arise from pre-existing epithelial dysplasia or from oral potentially malignant disorders (OPMDs) [1]. OPMDs correspond to alterations of the oral mucosa associated with an increased risk of developing oral cancer [2], among which oral leukoplakia (OL) and oral lichen planus (OLP) are the most relevant [3].

Oral leukoplakia is defined as a white plaque of uncertain risk after exclusion of other known diseases, and presents an estimated malignant transformation rate ranging from 5.1% to 13% [3-5]. In contrast, oral lichen planus is a chronic mucocutaneous disease with a reported malignant transformation rate between 1.1% and 2.3% [6,7]. These disorders develop within the context of field cancerization, a phenomenon in which clinically normal adjacent mucosa may harbor genetic alterations that favor malignant transformation [8].

Although current management protocols focus on the removal of morphologically altered areas, this approach does not completely eliminate the risk of OSCC development [9]. Therefore, non-surgical therapies aimed at achieving less invasive and better-tolerated treatments have been investigated. Among these, curcumin [a natural polyphenol derived from turmeric (1,7-bis(4-hydroxy-3-methoxyphenyl)-1,6-heptadiene-3,5-dione)] has been evaluated in clinical studies through both oral and topical administration [10].

Curcumin exhibits antioxidant, anti-inflammatory, antimicrobial, and antineoplastic properties, and has been shown to inhibit chemically induced carcinogenesis in organs such as the skin, stomach, and colon [11]. In addition, it has demonstrated a favorable safety profile, even at high doses [12]. However, evidence regarding its effects in patients with OPMDs remains limited. Therefore, turmeric and its derivatives may represent a promising therapeutic alternative that warrants further investigation.

The aim of this systematic review was to evaluate the available evidence on the clinical, anti-inflammatory, and molecular effects of curcumin in oral potentially malignant disorders and oral squamous cell carcinoma.

## Materials and Methods

This systematic review was conducted in accordance with the recommendations of the Cochrane Handbook for Systematic Reviews of Interventions and followed the four-phase flow diagram of the Preferred Reporting Items for Systematic Reviews and Meta-Analyses (PRISMA) statement.

The literature search was performed by the reviewers between September and December 2025 in the following databases: MEDLINE (National Library of Medicine, via PubMed), Web of Science, and SciELO. In addition, a manual search of the reference lists of the selected articles was carried out to identify potentially relevant studies. Articles published in English, Spanish, or Portuguese within the previous 15 years were considered. The search strategy was based on a combination of MeSH terms and keywords related to curcumin and oral potentially malignant disorders, including curcumin, Curcuma longa, turmeric, and turmeric extract, combined with terms related to oral leukoplakia, oral lichen planus, oral potentially malignant disorders, oral cancer, and oral squamous cell carcinoma.

Studies published in English, Portuguese, or Spanish that met the population, intervention, comparator, and outcomes (PICO) criteria were included. The population comprised adult patients diagnosed with oral leukoplakia, oral lichen planus, or oral squamous cell carcinoma. The intervention consisted of curcumin or Curcuma longa derivatives administered through any route, including oral and topical formulations, gels, and mouthwashes. Comparators included placebo, no treatment, or conventional therapies. The outcomes evaluated comprised clinical efficacy, anti-inflammatory effects, and changes in molecular biomarkers associated with curcumin use in these conditions.

Clinical trials, cohort studies, case-control studies, and observational studies conducted in humans were included. Animal studies, *in vitro* studies, narrative reviews, literature reviews, letters to the editor, and case reports were excluded.

After retrieval of the search results, titles and abstracts were screened independently by three reviewers using EndNote for duplicate removal and Rayyan® for the study selection process. Potentially eligible articles were subsequently assessed in full text to confirm compliance with the eligibility criteria.

The following data were extracted from each study: Authors and year of publication, study design, sample characteristics (country, number of patients, age, and gender), type of oral potentially malignant disorder evaluated, type of curcumin intervention (formulation, dose, and route of administration), reported clinical, anti-inflammatory, and molecular outcomes, and the main conclusions.

The methodological quality of the included studies was assessed using The Cochrane Risk of Bias 2 (RoB 2) tool was used for clinical trials, whereas the Newcastle-Ottawa Scale (NOS) was applied to observational studies. Owing to the high degree of heterogeneity among the included studies in terms of design, methodology, and analyzed variables, a quantitative meta-analysis was not considered appropriate; therefore, a qualitative synthesis of the results was performed.

## Results

A total of 1,527 records were identified through the electronic databases searched, with an additional three records identified through other sources. After removal of 301 duplicates and application of a 15-year time filter, 1,229 titles and abstracts were screened. Of these, 44 articles met the eligibility criteria and were selected for full-text review. Following detailed assessment and exclusion of studies with insufficient information, 18 articles were ultimately included in the qualitative synthesis [13-30] (Figure 1).

All selected studies were published in English between 2010 and 2024. Regarding geographic origin, nine studies were conducted in India (50.0%), four in Iran (22.2%), four in the United States (22.2%), and one in Belgium (5.6%). In terms of study design, the included studies comprised nine randomized controlled clinical trials, six case-control studies, two cross-sectional studies, and one cohort study.

Across the 18 included articles, sample sizes ranged from 10 to 300 participants, with a total of 1,189 individuals evaluated. Of these, 1,140 had a diagnosis of oral potentially malignant disorder (OPMD) or oral squamous cell carcinoma (OSCC), whereas 49 were controls. Among studies reporting participant age, the age range was 16 to 82 years. In studies that reported gender distribution, 428 men (54.5%) and 357 women (45.5%) were included. With respect to risk factors, five articles assessed tobacco and/or alcohol use. In these studies, tobacco smoking was reported in percentages ranging from 45.0% to 50.2%, tobacco chewing from 69.0% to 100%, and alcohol consumption from 36.7% to 41.7%. [13-30]. Regarding the clinical entities assessed, two studies included more than one OPMD, incorporating cases of oral leukoplakia (OL), oral lichen planus (OLP), and oral submucous fibrosis. Four studies specifically evaluated OL, ten focused on OLP, and two on OSCC. Overall, 621 cases of OL (54.5%), 465 cases of OLP (40.8%), 27 cases of oral submucous fibrosis (2.4%), and 27 cases of OSCC (2.4%) were recorded [13-30]. These data are summarized in Table 1. 

Among the studies evaluating oral leukoplakia, all four reported clinical improvement following curcumin administration, mainly reflected by lesion size reduction [13-16]. However, three of these studies did not observe significant histopathological changes after treatment. In one study, significant differences were identified in serum superoxide dismutase (SOD) levels in patients treated with nanocurcumin; the increase in this biomarker after therapy was accompanied by a decrease in lesion size [16].

With regard to oral lichen planus, eight of the ten included studies described improvement in clinical symptoms, particularly pain, burning sensation, and lesion size reduction. Studies assessing pain using the visual analog scale (VAS) showed relevant reductions after curcumin treatment [17,18,20-26]. For example, pain decreased from a baseline VAS score of 4.65 to 2.69 after intervention [17], whereas another study reported a reduction from 6.5 ± 2.15 to 0.33 ± 0.65 [18]. Similarly, one study reported a decrease from 3.75 to 2.88 (*p=*0.002) [23], while another described a reduction from 2.14 to 0.21 (*p=*0.001) [26]. Overall, these findings indicate a consistent trend toward pain reduction following curcumin administration, although some studies did not identify statistically significant differences compared with other treatments.

Five studies compared the efficacy of curcumin with corticosteroids; of these, four reported similar outcomes between both treatments, with no significant differences in the evaluated clinical parameters [17,18,21,22].

Regarding lesion size in patients with OLP, some studies reported clinically relevant reductions following curcumin administration. For example, one study observed a mean reduction from 3.83cm² to 2.34cm² (*p<*0.001) [17]. Similarly, another study reported a decrease from 2.34cm² to 0.27cm² (*p=*0.001) [26]. Although in other studies these differences did not reach statistical significance compared with comparator treatments, the overall trend supported lesion size reduction after curcumin intervention.

In the studies that included more than one OPMD [27,28], both investigations evaluated patients with OL, OLP, and oral submucous fibrosis and reported favorable outcomes following curcumin administration. These studies described significant reductions in pain and lesion size in patients with OLP, OL, and oral submucous fibrosis. In the latter condition, improvement in mouth opening was also reported.

Regarding the studies addressing OSCC, one study reported a significant decrease in the concentration of inflammatory cytokines, including TNF-α, IL-1β, and IL-8 [29], together with a reduction in the abundance of potentially pathogenic salivary microbiota, particularly the genera Fusicatenibacter, Dorea, Dialister, and Ruminococcus.

The route of curcumin administration varied across the included studies. Eleven studies used oral or systemic administration (61.1%), mainly in the form of capsules, tablets, or extracts. Seven studies employed topical administration (38.9%), including oral gels, toothpaste, mouthwashes, or microgranular formulations. In addition, in six studies, curcumin was administered as part of a combined therapy with other compounds or excipients, the characteristics of which are described in Table 2. Parameters related to pain assessment and changes in lesion size are presented in Table 3 and Table 4, respectively. Parameters and values of articles with evaluation of molecular markers or other variables are presented in Table 5.

**Discussion** 

Based on the available evidence, this review examined the clinical, anti-inflammatory, and molecular effects of curcumin in patients with oral leukoplakia (OL), oral lichen planus (OLP), and oral squamous cell carcinoma (OSCC). Most of the included studies were randomized clinical trials, which are regarded as high-level evidence, thereby strengthening the validity of the findings.

Most of the analyzed studies were conducted in India, which is consistent with the high burden of oral potentially malignant disorders (OPMDs) in this region. According to the International Agency for Research on Cancer, a substantial proportion of oral cancer cases worldwide occur in South Asia, with India representing one of the most heavily affected countries. This high prevalence has been linked to the widespread use of risk-related habits, including tobacco chewing, areca nut consumption, and alcohol intake [31].

From a biological perspective, curcumin exerts anti-inflammatory effects through the modulation of multiple molecular targets involved in inflammatory signaling. It has been shown to inhibit key enzymes such as cyclooxygenase-2 (COX-2) and 5-lipoxygenase (5-LOX), while also reducing the production of prostaglandins and proinflammatory cytokines, including IL-1, IL-2, IL-6, and tumor necrosis factor alpha (TNF-α) [32,33]. At the intracellular level, curcumin has been shown to modulate key inflammatory pathways, including NF-κB signaling, and to influence mediators associated with chronic inflammation and tissue remodeling. These effects have been associated with reductions in systemic inflammatory biomarkers, such as high-sensitivity C-reactive protein (hs-CRP), supporting its potential as an anti-inflammatory agent. [33].

In oral leukoplakia, the available studies indicate that curcumin is effective in reducing lesion size; however, no significant histopathological changes have been demonstrated following treatment [15]. This suggests that its clinical benefit may occur primarily at the symptomatic or macroscopic level, without necessarily translating into substantial changes in tissue architecture or in the biological behavior of the lesion.

Recent advances in the molecular characterization of OPMDs have highlighted the biological heterogeneity of oral leukoplakia, including the identification of distinct molecular and immune-related profilesm [34]. Transcriptomic analyses have identified distinct molecular subtypes within oral premalignant lesions, including “classical” and “immune-related” subtypes characterized by differential gene expression patterns and enrichment of immune regulatory pathways [35].

These findings raise the possibility that, in some cases, the biology of OL may be influenced by dysfunctional immune microenvironments characterized by a dysregulated or insufficient immune response unable to restrain the clonal progression of altered cells. In this context, curcumin emerges as a potentially relevant therapeutic alternative because of its ability to modulate key inflammatory processes. Its anti-inflammatory action, mediated by inhibition of proinflammatory cytokines and enzymes involved in the inflammatory cascade, together with its effects on oxidative stress, may contribute to partially restoring immune balance within the local tissue microenvironment. This mechanism may explain, at least in part, the reduction in lesion size observed clinically, particularly in lesions whose behavior is influenced by alterations in the local immune response.

Oral lichen planus is an immunopathological disorder in which CD8+ T lymphocytes recognize antigens expressed by basal keratinocytes and trigger a cytotoxic response, leading to epithelial damage and apoptosis [36]. In this context, curcumin treatment has been associated with symptomatic improvement. Furthermore, erosive and atrophic forms of oral lichen planus, often associated with more severe clinical presentation, have been linked to a higher risk of malignant transformation [2]. In this regard, some studies included in this review [reported clinical reductions in pain and burning sensation following curcumin use, suggest that curcumin may indirectly contribute to lowering the risk of progression to oral cancer, although this hypothesis requires confirmation in longitudinal studies.

Chronic inflammation exposes the oral epithelium to proinflammatory cytokines such as IL-6 and TNF-α, promoting oxidative stress and contributing to the progression of premalignant lesions [37]. Inflammatory cells within the tumor microenvironment can promote tumor progression through the production of growth factors, survival signals, angiogenic factors, and reactive oxygen species, as well as through extracellular matrix remodeling [38]. In this context, one of the included studies showed that curcumin reduced inflammatory markers in patients with OSCC. Elevated salivary levels of proinflammatory cytokines, particularly IL-1β and IL-8, were observed at baseline and decreased after treatment. These findings suggest that curcumin may attenuate tumor-related processes by reducing proinflammatory cytokines [29].

Among the main limitations of this review is the methodological heterogeneity of the included studies, particularly with regard to the doses administered, the formulations used (such as capsules, gels, or nanoparticles) the duration of treatment, and the routes of administration. This heterogeneity hinders direct comparison of results and precludes identification of a clearly superior formulation. In addition, there was substantial variability in the outcomes assessed and in the way results were reported, as some studies focused exclusively on clinical parameters, whereas others included histopathological evaluation, inflammatory biomarkers, or subjective measures of improvement. This diversity in endpoints, measurement units, and assessment methods limits robust synthesis of the findings and reduces the generalizability of the conclusions.

Despite these limitations, the available evidence suggests that curcumin has relevant therapeutic potential across different OPMDs, likely related to its ability to modulate inflammatory, oxidative, and proliferative pathways involved in carcinogenesis. From a clinical standpoint, curcumin has been associated with reduced lesion size in OL and with a consistent reduction in pain and burning sensation in OLP. In OSCC, although the evidence remains limited, curcumin may represent a potential adjunctive therapy because of its ability to reduce inflammatory mediators and contribute to a microenvironment less favorable to tumor progression.

## Conclusions

Taken together, these findings support curcumin as a promising adjunctive therapeutic option, particularly in the management of OLP, in which its clinical efficacy has been reported to be comparable to that of conventional therapies in some studies. However, the lack of consistent evidence regarding histopathological changes, together with the methodological heterogeneity of the included studies, limits the strength of these conclusions. Therefore, although curcumin appears to have a favorable safety profile and relevant therapeutic potential, its incorporation into clinical practice should be approached with caution. Further well-designed controlled clinical trials with larger sample sizes, standardized doses and formulations, and homogeneous outcome measures are needed to more precisely define its efficacy, optimal dosage, and potential role in preventing malignant transformation. 

Curcumin appears to have favorable clinical and anti-inflammatory effects in oral leukoplakia, oral lichen planus, and oral squamous cell carcinoma, mainly reflected by reductions in pain and lesion size. However, the heterogeneity of the available studies and the lack of consistent histopathological evidence limit the strength of these findings. Further well-designed clinical trials are needed to better define its efficacy, optimal dosage, and potential role in clinical practice. 

## Figures and Tables

**Table 1:** Articles included in the Systematic Review.

**Author**	**Country**	**Diagnosis**	**Study type**	**Cases (n)**	**Controls (n)**	**Age**	**Gender**		**Habits**	
							**Male**	**Female**	**Smoke**	**Alcohol**
Ahmad T *et al*., 2023	India	OL	Clinical Trial	300	-	18-70	N/S		0	0
Kuriakose MA *et al*., 2016	India	OL	Case Control	223	112	21-75	161	62	112	93
Khare P *et al*., 2024	India	OL	Cross-sectional study	10	-	18-65	10	0	10	10
Deb S, *et al*., 2022	India	OL	Cross-sectional study	30	-	18-65	30	0	N/S	
Kia SJ *et al*., 2020	Iran	OLP	Clinical Trial	50	-	38-73	14	36	N/S	
Amirchaghmaghi M *et al*., 2016	Iran	OLP	Clinical Trial	20	-	N/S	7	13	N/S	
Chainani-Wu N, 2012	EEUU	OLP	Case Control	20	10	N/S	10	10	N/S	
Chainani-Wu N, 2012	EEUU	OLP	Cohort	53	16	N/S	N/S		N/S	
Kia SJ *et al*., 2015	Iran	OLP	Clinical Trial	60	-	N/S	9	48	N/S	
Nosratzehi T, *et al*., 2017	Iran	OLP	Clinical Trial	40	-	28-60	14	26	N/S	
Khaitan T, *et al*., 2022	India	OLP	Clinical Trial	40	-	>20	14	26	N/S	
Srivastava R *et al*., 2024	India	OLP	Case Control	15	15	N/S	7	23	N/S	
Thomas AE, *et al*., 2017	India	OLP	Clinical Trial	75	-	N/S	19	56	N/S	
Shivu ME *et al*., 2024	India	OLP	Clinical Trial	30	-	N/S	N/S		N/S	
Neetha MC *et al*., 2020	India	OPMD*	Clinical Trial	60	-	16-82	45	15	27	22
Rai B, *et al*., 2010	Belgium	OPMD*	Case Control	75	25	17-50	36	39	0	0
Basak SK *et al*., 2020	EEUU	HNSCC	Case Control	12	13	>18	24	1	12	N/S
Latimer B *et al*., 2015	EEUU	HNSCC	Case Control	15	8	22-76	14	1	N/S	

OL: Oral leukoplakia, OLP: Oral lichen planus, OPMD*: Oral potentially malignant disorders (these include oral lichen planus, oral leukoplakia, and submucosal oral fibrosis), HNSCC: Head and neck squamous cell carcinoma, N/S: Not specify.

**Table 2:** Characterization and results of selected articles.

**Author**	**Diagnosis**	**Route of Administration**	**Posology**	**Parameters evaluated**	**Result**	*P*
Ahmad T *et al*., 2023	OL	Oral / Systemic	Curcumin 500mg/day Lycopene 4mg/day Curcumin 500mg + Lycopene 4mg/day	Lesion size Lesion color	Curcumin and lycopene in combination is effective for treating OL.	*p<*0,05*
Kuriakose MA *et al*., 2016	OL	Oral / Systemic	Curcumin 3,6mg/day Placebo	Lesion size	Curcumin is clinically effective for treating OL; however, no improvement was observed at the histological level.	*p<*0,05*
Khare P *et al*., 2024	OL	Oral / Systemic	Curcumin	Histological analysis	Curcumin is effective for controlling symptoms in patients with OL, but not at the histological level.	N/S
Deb S, *et al*., 2022	OL	Oral / Systemic	Curcumin 250mg/day	Superoxide dismutase level (SOD)	Curcumin caused an increase in serum superoxide dismutase levels in patients with OL.	*p<*0,05*
Kia SJ *et al*., 2020	OLP	Oral / Systemic	Curcumin 80mg/day Prednisolone 10mg	Pain reduction Lesion size	Curcumin is similarly effective as oral corticosteroids in reducing pain and lesion size for the treatment of OLP.	*p>*0,05
Amirchaghmaghi M *et al*., 2016	OLP	Oral / Systemic	Curcumina 2000mg/día + Dexamethasone 0.5mg + Nystatin 100,000 Placebo + Dexamethasone 0.5mg + Nystatin 100,000	Pain reduction Thangprasom score	Curcumin showed no difference compared to the placebo group when used in conjunction with steroid treatment for the treatment of OLP.	*p>*0,05
Chainani-Wu N, 2012	OLP	Oral / Systemic	Curcumin 6000mg/day Placebo	Modified Oral Mucositis Index (MOMI) C-reactive protein Interleukin-6	Curcumin 6000mg/day orally is effective for reducing the signs and symptoms of lesions in patients with OLP.	*p<*0,05*
Chainani-Wu N, 2012	OLP	Oral / Systemic	Capsule curcumin 2000mg/day Capsule curcumin 6000mg/day	Perception of symptom relief	A total of 22/37 (60%) patients reported a reduction in symptoms of OLP lesions after treatment with oral curcumin.	N/S
Kia SJ *et al*., 2015	OLP	Topical	Curcumin 5% paste Triamcinolone 0.1% paste	Pain reduction Lesion size	Curcumin shows similar effectiveness in reducing pain and lesion size as topical corticosteroids for the treatment of OLP.	*p>*0,05
Nosratzehi T, *et al*., 2017	OLP	Topical	Curcumin paste Corticosteroids paste	Pain reduction Severity of the injury Lesion size	Curcumin shows similar effectiveness in reducing pain and lesion severity as topical corticosteroids for the treatment of OLP	*p>*0,05
Khaitan T, *et al*., 2022	OLP	Topical	Curcumin 10mg gel + Zinc Zinc	Pain reduction Severity of the injury	Curcumin in gel form is effective for reducing pain and severity of lesions in patients with OLP	*p<*0,05*
Srivastava R et el., 2024	OLP	Topical	Curcumin 30mg gel + calendula + tulsi Placebo	Remission of signs and symptoms	Curcumin in gel form, in combination with calendula and tutsi, is effective for treating lesions in patients with OLP	*p<*0,05*
Thomas AE, *et al*., 2017	OLP	Topical	Triamcinolone acetonide 0,1% paste Curcumin 1% paste 1 per day Curcumin 1% paste 2 per day	Modified Oral Mucositis Index (MOMI)	In all three groups, a reduction was observed in pain sensation, lesion severity, and lesion size of OLP	*p<*0,05*
Shivu ME *et al*., 2024	OLP	Topical	Turmeric gel Ashwagandha gel Aloe vera gel	Pain reduction Burning sensation Lesion size	Curcumin in gel form used for 4 months is effective in reducing pain, burning sensation, and lesion size in patients with OLP	*p<*0,05*
Neetha MC *et al*., 2020	OPMD*	Oral / Systemic	Green Tea 800mg/day Curcumin 950mg/day Green Tea 800mg/día + Curcumin 950mg/day	Clinical response Histopathological Analysis (Ki-67, cyclin D1 and p53)	Curcumin in combination with green tea is effective for controlling the symptoms of the studied OPMDs, but not at the histological level	*p<*0,05*
Rai B, *et al*., 2010	OPMD*	Oral / Systemic	Curcumin 900mg 1 capsule	Malondialdehyde (MDA) 8-OhdG Vitamin C Vitamin E	Curcumin increases local and systemic antioxidant status and vitamin C and E levels, while decreasing lipid peroxidation and DNA damage in patients with OPDMs.	*p<*0,05*
Basak SK *et al*., 2019	HNSCC	Oral / Systemic	Curcumin 300mg 1 capsule per day Curcumin 300mg 2 capsule per day Placebo	Salivary cytokines (TNF-α, IL-1β, IL-8) Microbiome	A statistically significant reduction in the concentration of inflammatory cytokines and Bacteroides species was observed in salivary cells	*p<*0,05*
Latimer B *et al*., 2015	HNSCC	Topical	Curcumin 4000mg	Serum levels of curcumin FGF-2 GM-CSF IL-17	Curcumin administered via microgranulation increases serum curcumin levels in both cancer patients and healthy volunteers	*p<*0,05*

OL: Oral leukoplakia, OLP: Oral lichen planus, OPMD*: Oral potentially malignant disorders (these include oral lichen planus, oral leukoplakia, and submucosal oral fibrosis), HNSCC: Head and neck squamous cell carcinoma, 8-OhdG: 8-hidroxi-2'-desoxiguanosina, FGF-2: Fibroblast growth factor 2, GM-CSF: Granulocyte-macrophage colony-stimulating factor, IL-17: Interleukin 17, TNF-α: Tumor necrosis factor alpha, IL-1β: Interleukin-1 beta, IL-8: Interleukin 8, N/S: Not specify.

**Table 3:** Parameters and values of articles with pain assessment.

**Author**	**Diagnosis**	**Posology**	**Pain Reduction**		*p*
			**Initial**	**Final**	
Kia SJ *et al*., 2020	OLP	Curcumin 80mg/day Prednisolone 10mg	Curcumin EVA 4.65 Prednisolone EVA 4.89	Curcumin EVA 2.69 Prednisolone EVA 2.33	<0,001
Amirchaghmaghi M *et al*., 2016	OLP	Curcumin 2000mg/day + Dexamethasone 0.5mg + Nystatin 100,000 Placebo + Dexamethasone 0.5mg + Nystatin 100,000	Curcumin EVA 6.5 ± 2.15 Placebo EVA 4.63 ± 3.20	Curcumin EVA 0.33 ± 0.65 Placebo EVA 0.13 ± 0.35	0,002
Kia SJ *et al*., 2015	OLP	Curcumin 5% paste Triamcinolone 0.1% paste	Curcumin EVA 5.84 ± 2.01 Triamcinolone EVA 5.47 ± 3.12	Curcumin EVA 2.64 ± 2.98 Triamcinolone EVA 1.76 ± 1.78	0,46
Nosratzehi T, *et al*., 2017	OLP	Curcumin paste Corticosteroids paste	Curcumin EVA 1.91 ± 0.76 Corticosteroids EVA 1.85 ± 0.63	Curcumin EVA 0.38 ± 0.35 Corticosteroids EVA 0.41 ± 0.39	0,12
Khaitan T, *et al*., 2022	OLP	Curcumin 10mg gel + Zinc Zinc	Curcumin EVA 3.75 Zinc EVA 4.3	Curcumin EVA 2.88 Zinc EVA 1.24	0,002
Shivu ME *et al*., 2024	OLP	Tumeric gel Ashwagandha gel Aloe vera gel	Tumeric EVA 2.14 Ashwagandha EVA 2.17 Aloe vera EVA 2.19	Tumeric EVA 0.21 Ashwagandha EVA 0.27 Aloe vera EVA 0.31	0,001

**Table 4:** Parameters and values of articles with lesion size assessment

**Author**	**Diagnosis**	**Posology**	**Lesion size**		*p*
			**Initial**	**Final**	
Kia SJ *et al*., 2020	OLP	Curcumin 80mg/day Prednisolone 10mg	Curcumin 3.83cm² Prednisolone 3.61cm²	Curcumin 2.34cm² Prednisolone 1.83cm²	<0.001
Kia SJ *et al*., 2015	OLP	Curcumin 5% paste Triamcinolone 0.1% paste	Curcumin 3.88 ± 0.78cm² Triamcinolone 3.95 ± 1.07cm²	Curcumin 2.64 ± 1.29 cm² Triamcinolone 2.95 ± 0.97cm²	0,31
Nosratzehi T, *et al*., 2017	OLP	Curcumin paste Corticosteroids paste	Curcumin 2.45 ± 1.1cm² Corticosteroids 2.61 ± 1.2cm²	Curcumina 0.75 ± 0.34cm² Corticosteroids 0.91 ± 0.46cm²	0,66
Shivu ME *et al*., 2024	OLP	Tumeric gel Ashwagandha gel Aloe vera gel	Tumeric 2.34cm² Ashwagandha 2.36cm² Aloe vera 2.38cm²	Tumeric 0.27cm² Ashwagandha 0.35cm² Aloe vera 0.38cm²	0,001

**Table 5:** Parameters and values of articles with evaluation of molecular markers or other variables.

**Author**	**Diagnosis**	**Posology**	**Other Parameters/Results**		*p*
			**Initial**	**Final**	
Ahmad T *et al*., 2023	OL	Curcumin 500mg/day Lycopene 4mg/day Curcumin 500mg + Lycopeno 4mg/day	**Number of patients with favorable evolution **Curcumin n=51/94 (51%) Lycopene n=63/95 (63%) Curcumin + Lycopene n=88 (88%)		N/S
Kuriakose MA *et al*., 2016	OL	Curcumin 3,6mg/day Placebo	**Number of patients with favorable evolution **Curcumin n=65 (61,9%) Placebo n=50 (46,3%)		0,03
Deb S, *et al*., 2022	OL	Curcumin 250mg/day	SOD 148.89±23.63U/ml	SOD 170.38±14.08U/ml	<0,0001
Amirchaghmaghi M *et al*., 2016	OLP	Curcumin 2000mg/day + Dexamethasone 0.5mg + Nystatin 100,000 Placebo + Dexamethasone 0.5mg + Nystatin 100,000	Thangprasom Curcumin 3.17 ± 1.03 Placebo 3 ± 1.30	Thangprasom Curcumin 1.08 ± 0.66 Placebo 1.5 ± 1.06	0,002
Chainani-Wu N, 2012	OLP	Curcumin 6000mg/day Placebo	MOMI 9.5 PCR 0,25 IL-6 2.175	MOMI 7.75 PCR 0,4 IL-6 1.65	N/S
Nosratzehi T, *et al*., 2017	OLP	Curcumin paste Corticosteroids paste	**Severity of the injury **Curcumine stage 9.68 ± 2.95 Corticosteroids stage 3.05 ± 9.51	**Severity of the injury ** Curcumin stage 2.81 ± 2.41 Corticosteroids stage 2.3 ± 1.76	0,12
Khaitan T, *et al*., 2022	OLP	Curcumin 10 mg gel + Zinc Zinc	**Severity of the injury **Curcumin REU 4.49 Zinc REU 4.5	**Severity of the injury **Curcumin REU 1.36 Zinc REU 0.43	<0,001
Srivastava R et el., 2024	OLP	Curcumin 30 mg gel + calendula + tulsi Placebo	**Number of patients with favorable evolution** Curcumin n=9 (60,00%) Placebo n=4 (26,67%)		<0,001
Thomas AE, *et al*., 2017	OLP	Triamcinolone acetonide 0,1% paste Curcumin 1% paste once per day Curcumin 1% paste twice per day	MOMI 11.52 ± 1.63	MOMI 3.72 ± 2.22	<0,001
Shivu ME *et al*., 2024	OLP	Tumeric gel Ashwagandha gel Aloe vera gel	**Burning sensation **Tumeric EVA 2.12 Ashwagandha EVA 2.18 Aloe vera EVA 2.16	**Burning sensation **Tumeric EVA 0.24 Ashwagandha EVA 0.34 Aloe vera EVA 0.42	0,001
Rai B, *et al*., 2010	OPMD*	Curcumin 900mg 1 capsule	MDA 0.34umol/l 8-OhdG 0.35ng/m Vitamin C 1.04ug/l Vitamin E 0.66ug/l	MDA 0.12umol/l 8-OhdG 0.12ng/ml Vitamin C 1.51ug/l Vitamin E 0.92ug/l	<0,001
Latimer B *et al*., 2015	HNSCC	Curcumin 4000mg	FGF-2 90.4 ± 142.6pg/ml GM-CSF 30.7 ± 59.8pg/ml IL-17 29.4 ± 89.7pg/ml	FGF-2 40.8 ± 53.7pg/ml GM-CSF 19.4 ± 46.6pg/ml IL-17 5.3 ± 6.1pg/ml	<0,10

OL: Oral leukoplakia, OLP: Oral lichen planus, OPMD*: Oral potentially malignant disorders (these include oral lichen planus, oral leukoplakia, and submucosal oral fibrosis), HNSCC: Head and neck squamous cell carcinoma, 8-OhdG: 8-hidroxi-2'-desoxiguanosina, FGF-2: Fibroblast growth factor 2, GM-CSF: Granulocyte-macrophage colony-stimulating factor, IL-17: Interleukin 17, N/S: Not specify.

**Figure 1:**
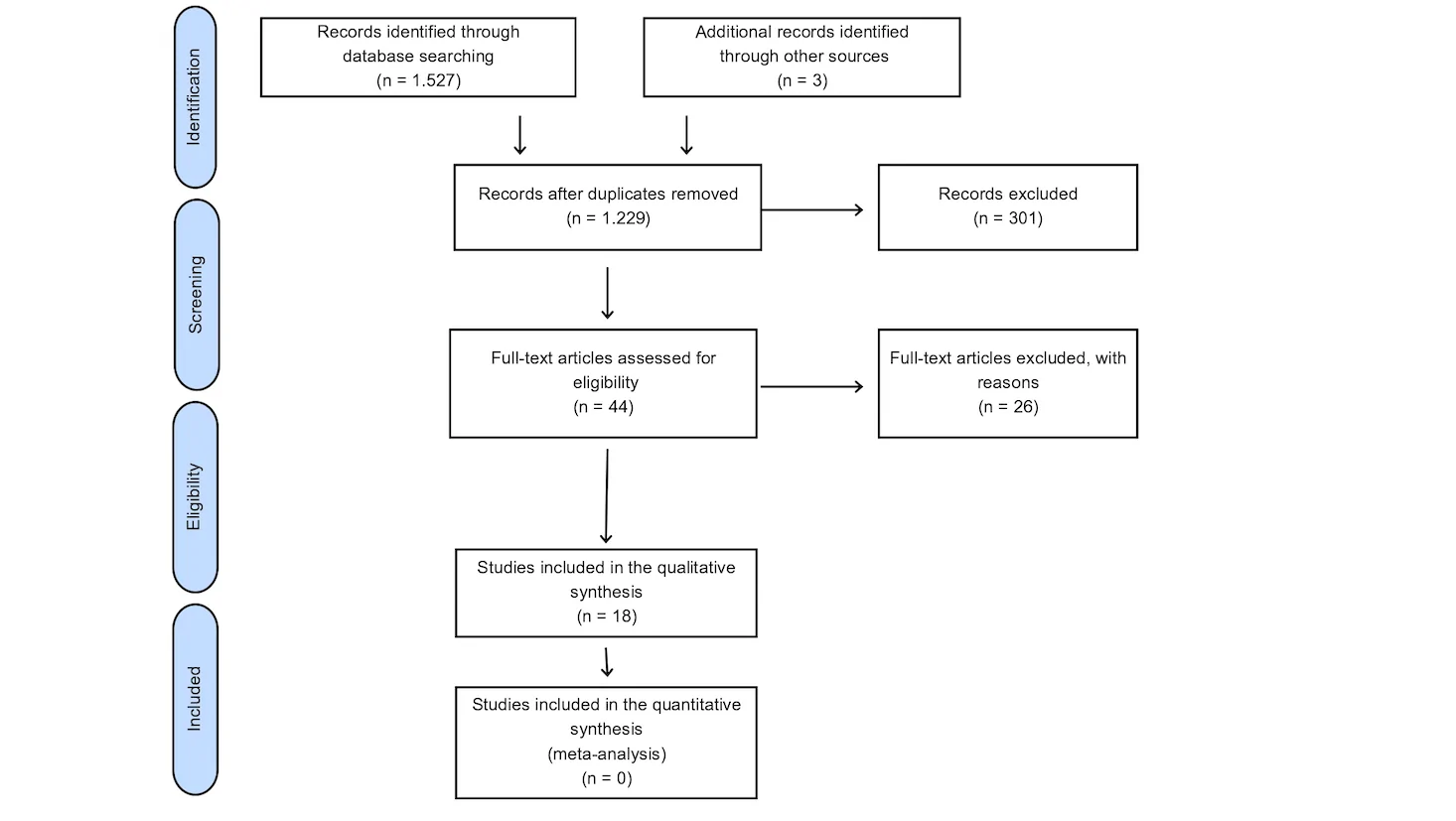
Search flow diagram according to PRISMA.

## Data Availability

The data supporting the findings of this study are available within the article and its supplementary materials.

## References

[1] WHO Classification of Tumours Editorial Board. Head and neck tumours. 5th ed. Vol. 9. Lyon: International Agency for Research on Cancer; 2022.

[2] Warnakulasuriya S, Kujan O, Aguirre-Urizar JM, Bagan JV, González-Moles MÁ, Kerr AR, Lodi G, Weber Mello F, Monteiro L, Ogden GR, Sloan P, Johnson NW (2021). Oral potentially malignant disorders: a consensus report from an international seminar on nomenclature and classification, convened by the WHO Collaborating Centre for Oral Cancer. Oral Dis.

[3] Warnakulasuriya S, Ramos-García P, González-Moles MÁ (2026). Oral lichen planus and systemic diseases. J Dent Res.

[4] Iocca O, Sollecito TP, Alawi F, Weinstein GS, Newman JG, De Virgilio A, Di Maio P, Spriano G, Pardiñas López S, Shanti RM (2020). Potentially malignant disorders of the oral cavity and oral dysplasia: a systematic review and meta-analysis of malignant transformation rate by subtype. Head Neck.

[5] Scully C, Bagan J (2009). Oral squamous cell carcinoma: an overview. Oral Oncol.

[6] González-Moles MÁ, Warnakulasuriya S, González-Ruiz I, González-Ruiz L, Ayén Á, Lenouvel D, Ruiz-Ávila I, Ramos-García P (2021). Worldwide prevalence of oral lichen planus: a systematic review and meta-analysis. Oral Dis.

[7] González-Moles MÁ, Warnakulasuriya S, González-Ruiz I, Ayén Á, González-Ruiz L, Ruiz-Ávila I, Ramos-García P (2021). Dysplasia in oral lichen planus: relevance, controversies and challenges. A position paper. Med Oral Patol Oral Cir Bucal.

[8] Sathiasekar AC, Mathew DG, Lal MSJ, Prakash A, Kumar KUG (2017). Oral field cancerization. J Pharm Bioallied Sci.

[9] Lodi G, Franchini R, Warnakulasuriya S, Varoni EM, Sardella A, Kerr AR, Carrassi A, MacDonald LCI, Worthington HV. Interventions for treating oral leukoplakia to prevent oral cancer. Cochrane Database Syst Rev. 2016;(7):CD001829. 10.1002/14651858.CD001829.pub4 PMC6457856 27471845

[10] Khosrojerdi M, Gumpricht E, Jamialahmadi T, Kesharwani P, Sahebkar A (2023). Curcumin in oral lichen planus. Curr Med Chem.

[11] Iranshahi M, Sahebkar A, Hosseini ST, Takasaki M, Konoshima T, Tokuda H (2010). Cancer chemopreventive activity of diversin. Phytomedicine.

[12] Soleimani V, Sahebkar A, Hosseinzadeh H (2018). Turmeric and curcumin: a review of therapeutic and biological activities. Phytother Res.

[13] Ahmad T, Khan I, Sultana A, Rizvi M, Moshahid A (2023). A comparative study to evaluate the therapeutic effects of nutraceuticals in oral leukoplakia: a randomized clinical trial. Natl J Maxillofac Surg.

[14] Kuriakose MA, Ramdas K, Dey B, Iyer S, Rajan G, Elango KK, Suresh A, Ravindran D, Kumar RR, Prathiba R, Ramachandran S, Asok Kumar N, Thomas G, Somanathan T, Ravindran HK, Ranganathan K, Katakam SB, Parashuram S, Jayaprakash V, Pillai MR (2016). A randomized double-blind placebo-controlled phase IIB trial of curcumin in oral leukoplakia. Cancer Prev Res (Phila).

[15] Khare P, Devra AG, Patidar M, Punga R, Gupta N, Sohn SY (2024). Curcumin lozenges in leukoplakia. J Oral Maxillofac Pathol.

[16] Deb S, Bhargava D, Bansal P, Kanuru V (2022). Role of nano curcumin on superoxide dismutase levels in leukoplakia. J Oral Maxillofac Pathol.

[17] Kia SJ, Basirat M, Mortezaie T, Moosavi MS (2020). Oral nano-curcumin versus prednisolone in oral lichen planus. BMC Complement Med Ther.

[18] Amirchaghmaghi M, Pakfetrat A, Delavarian Z, Ghalavani H, Ghazi A. Evaluation of the efficacy of curcumin in the treatment of oral lichen planus: a randomized controlled trial. J Clin Diagn Res. 2016;10(5):ZC134-7. 10.7860/JCDR/2016/16338.7870 PMC4948524 27437348

[19] Chainani-Wu N, Madden E, Lozada-Nur F, Silverman S Jr. High-dose curcuminoids are efficacious in reducing symptoms and signs of oral lichen planus. J Am Acad Dermatol. 2012;66(5):752-60. 10.1016/j.jaad.2011.04.022 21907450

[20] Chainani-Wu N, Collins K, Silverman S Jr. Use of curcuminoids in a cohort of patients with oral lichen planus. Phytomedicine. 2012;19(5):418-23. 10.1016/j.phymed.2011.11.005 22305276

[21] Kia SJ, Shirazian S, Mansourian A, Khodadadi Fard L, Ashnagar S (2015). Comparative efficacy of topical curcumin and triamcinolone. J Dent (Tehran).

[22] Nosratzehi T, Arbabi-Kalati F, Hamishehkar H, Bagheri S (2018). Curcumin mucoadhesive paste in oral lichen planus. J Natl Med Assoc.

[23] Khaitan T, Vishal, Kabiraj A, Sinha DK, Ranjan R, Singh R. Curcuma longa in the treatment of symptomatic oral lichen planus. Indian J Dermatol. 2022;67(4):478. 10.4103/ijd.ijd_1065_20 PMC9792051 36578734

[24] Srivastava R, Sarkar S, Gupta P, Pradhan D, Bagde H, Dhopte A (2024). Ayurvedic gel in erosive oral lichen planus. J Pharm Bioallied Sci.

[25] Thomas AE, Varma B, Kurup S, Jose R, Chandy ML, Kumar SP, Aravind MS, Ramadas AA. Evaluation of efficacy of 1% curcuminoids as local application in management of oral lichen planus: interventional study. J Clin Diagn Res. 2017;11(4):ZC89-93. 10.7860/JCDR/2017/20898.9715 PMC5449927 28571271

[26] Shivu ME, Gupta N, Mulla M, Mulla M, Bajoria AA, Varshney A (2024). Evaluation of turmeric, ashwagandha, and aloe vera in the management of oral lichen planus lesion. J Pharm Bioallied Sci.

[27] Neetha MC, Panchaksharappa MG, Pattabhiramasastry S, Shivaprasad NV, Venkatesh UG (2020). Synergism between green tea extract and curcumin. J Contemp Dent Pract.

[28] Rai B, Kaur J, Jacobs R, Singh J (2010). Mechanism of curcumin in pre-cancerous lesions. J Oral Sci.

[29] Basak SK, Bera A, Yoon AJ, Morselli M, Jeong C, Tosevska A, Dong TS, Eklund M, Russ E, Nasser H, Lagishetty V, Guo R, Sajed D, Mudgal S, Mehta P, Avila L, Srivastava M, Faull K, Jacobs J, Pellegrini M, Shin DS, Srivatsan ES, Wang MB (2020). A randomized, phase 1, placebo-controlled trial of APG-157 in oral cancer demonstrates systemic absorption and an inhibitory effect on cytokines and tumor-associated microbes. Cancer.

[30] Latimer B, Ekshyyan O, Nathan N, Moore-Medlin T, Rong X, Ma X, Khandelwal A, Christy HT, Abreo F, McClure G, Vanchiere JA, Caldito G, Dugas T, McMartin K, Lian T, Mehta V, Nathan CAO (2015). Enhanced systemic bioavailability of curcumin through transmucosal administration of a novel microgranular formulation. Anticancer Res.

[31] Sridharan G (2024). Areca nut and tobacco chewing and oral cancer: a review. Indian J Med Paediatr Oncol.

[32] Basnet P, Skalko-Basnet N (2011). Curcumin: an anti-inflammatory molecule from a curry spice on the path to cancer treatment. Molecules.

[33] Salehi B, Stojanović-Radić Z, Matejić J, Sharifi-Rad M, Kumar NVA, Martins N, Sharifi-Rad J (2019). The therapeutic potential of curcumin: a review of clinical trials. Eur J Med Chem.

[34] Farah CS (2021). Molecular, genomic and mutational landscape of oral leukoplakia. Oral Dis.

[35] Foy JP, Bertolus C, William WN Jr, Saintigny P. Immunological and classical subtypes of oral premalignant lesions. Oncoimmunology. 2018;7(10):e1496880. 10.1080/2162402X.2018.1496880 PMC6279331 30524889

[36] Kurago ZB (2016). Etiology and pathogenesis of oral lichen planus: an overview. Oral Surg Oral Med Oral Pathol Oral Radiol.

[37] Khamis A, Salzer L, Schiegnitz E, Stauber RH, Gül D (2023). Vitamin D and oral potentially malignant disorders. Int J Mol Sci.

[38] Hanahan D, Weinberg RA (2011). Hallmarks of cancer: the next generation. Cell.

